# Size-dependent bioactivity of electrosprayed core–shell chitosan-alginate particles for protein delivery

**DOI:** 10.1038/s41598-022-24389-x

**Published:** 2022-11-22

**Authors:** Sayna Shamszadeh, Mohammad Akrami, Saeed Asgary

**Affiliations:** 1 Iranian Center for Endodontic Research, Research Institute of Dental Sciences, Shahid Beheshti University of Medical Sciences, Tehran, 1983963113 Iran; 2 Department of Pharmaceutical Biomaterials and Medical Biomaterials Research Center, Faculty of Pharmacy, Tehran University of Medical Sciences, Tehran, 1417614411 Iran; 3 Institute of Biomaterials, University of Tehran and Tehran University of Medical Sciences (IBUTUMS), Tehran, Iran

**Keywords:** Drug delivery, Nanoparticles

## Abstract

Nano-bio interactions are size-dependent. The present study investigates whether core–shell chitosan-alginate particle size governs biological activities as well as protein release profile. A coaxial electrospraying was used to fabricate bovine serum albumin (BSA)-loaded core–shell micro/nanoparticles and were fully characterized. The bio/hemocompatibility of the particles was assessed using MTT and hemolytic assays, respectively, followed by the uptake assessment using flow cytometry. Finally, protein absorption was investigated using SDS-PAGE. The SEM size of the microparticles, the hydrodynamic, and the actual sizes of the nanoparticles were 1.2 μm, 90.49 nm, and 50 nm, respectively. Interactions among two polymers and BSA were observed using DSC analysis. BET analysis showed a more surface area for nanoparticles. A sustained release trend of BSA was observed after 14- and 10-day for microparticles and nanoparticles, respectively. Microparticles exhibited excellent hemocompatibility (< 5% hemolysis) and cell viability (at least > 70%) in all concentrations. However, acceptable hemolytic activity and cell viability were observed for nanoparticles in concentrations below 250 μg/mL. Furthermore, nanoparticles showed greater cellular uptake (~ 4 folds) and protein absorption (~ 1.61 folds) than microparticles. Overall, the developed core–shell chitosan-alginate particles in the micro/nanoscale can be promising candidates for biomedical application and regenerative medicine regarding their effects on above mentioned biological activities.

## Introduction

Over recent years, the increasing use of therapeutic proteins, such as growth factors, has been reported in regenerative medicine. However, the direct administration of therapeutic proteins is limited due to their short half-life and susceptibility to protease degradation^1^. Repeated dose administration is often required to achieve physiological and pharmacodynamic effects. Therefore, approaches that achieve controlled-release delivery and prevent degradation are extremely beneficial^2^.

Numerous techniques have been settled to prepare protein delivery systems, such as emulsification solvent evaporation^3^ or spray drying^4^, but most of these polymer-based methods have some disadvantages, including low encapsulation efficiency, wide particle size distribution, and high initial burst release^3,5^. In addition, when proteins are exposed to harsh environmental conditions during the manufacturing process (organic solvents, changes in temperature, and shear stress), their structure can be modified, which leads to chemical degradation (e.g., fragmentation, hydrolysis, or oxidation) and physical instability (aggregation, precipitation, conformational changes, or denaturation)^6^. As an alternative approach, electrospraying has recently shown great potential for producing a protein-loaded vehicle with relatively narrow particle size distribution and intact biological activity^7^. This is actually reasonable because the biological macromolecules not only will be protected inside biocompatible polymeric matrixes, but also will be released controllably and sustainably at a specific site.

In the regeneration process, it has been reported that multiple growth factors are expressed sequentially^8^. Core–shell particles have emerged recently as promising vehicles for sustained drug delivery in order to deliver multiple biomolecules sequentially, avoid the initial burst release, and adjust the drug release rate^9^. The coaxial electrospray technique has gained considerable attention in synthesizing biodegradable polymeric particles with core–shell structures^10,11^.

As biocompatible and biodegradable polymers, chitosan^12^ and alginate^13^ are known as high potential carriers in pharmaceutical fields. Fabrication of the positively charged chitosan and negatively charged alginate into a bilayer core–shell structure could be efficient and cost-effective^14^.

The particle size is another parameter that can influence the core–shell particles' release kinetics and biological fate. It has been reported that reducing the bulk scale to the nanometer range can improve the delivery properties of bioactive compounds^15^. As a delivery system, nanoparticles have several advantages (i.e., the greater surface to volume ratio and intracellular uptake) compared to microspheres^16^. In addition, nanospheres usually cause little or no local inflammation, while microspheres in size range of 5–10 µm produce prominent inflammatory reactions with subsequent fibrosis^17^. Instead, many studies have shown that microspheres have much better drug capacity and sustained drug release properties^18,19^.

Thus, this study aims to investigate the effect of micro/nanoscale particles on their protein loading, release profiles, and biological fate. In this regard, protein-loaded micro/nano core–shell chitosan-alginate particles were fabricated using coaxial electrospray methods. The particles were characterized by size, release profile, hemo/bio-compatibility, protein absorption, and cellular uptake.

## Results

**Figure 1:**
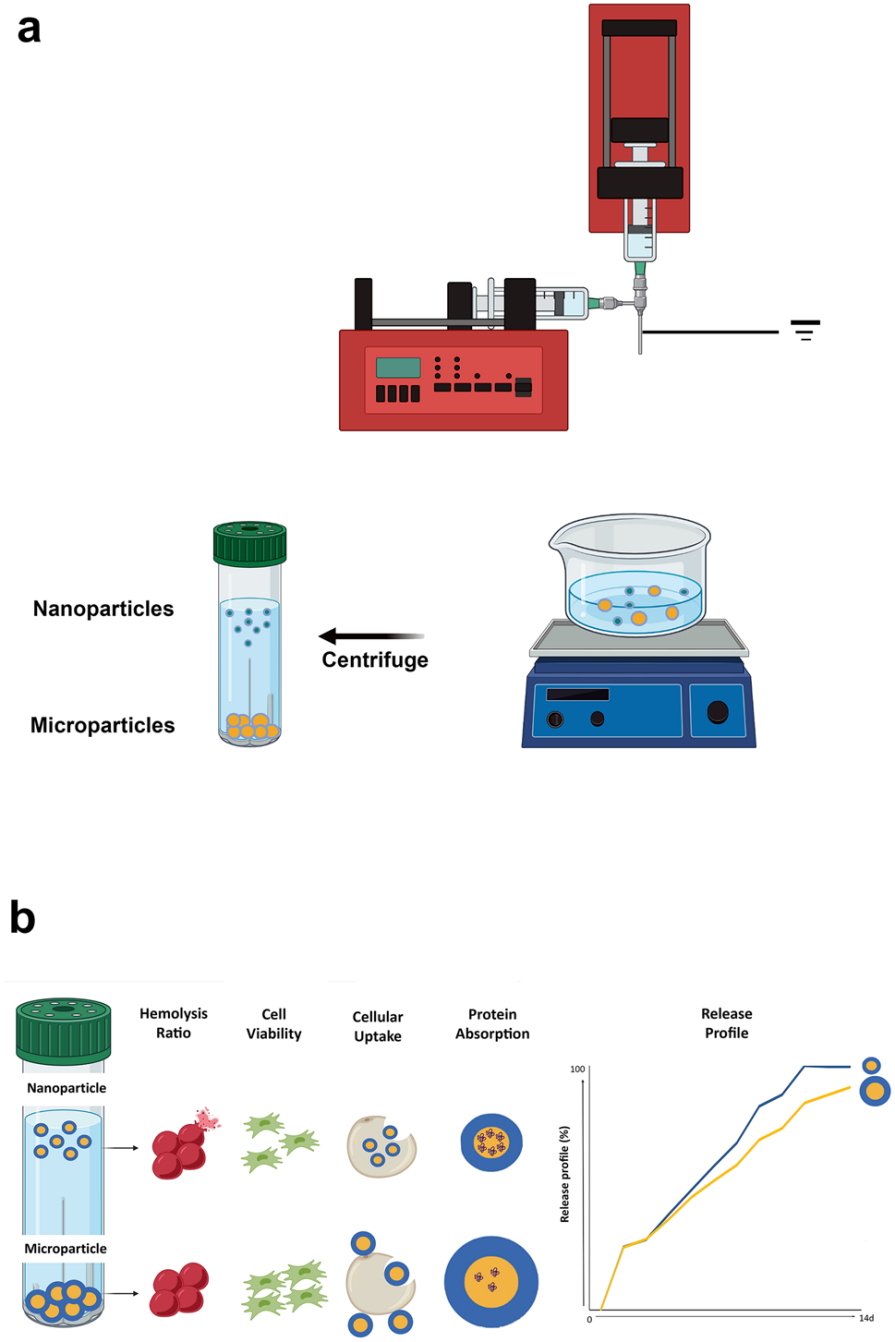
Experimental setup for electrospray fabrication of chitosan-alginate core–shell particles, and (**b**) schematic represents the bioactivity of chitosan-alginate core–shell particles.

The steps for preparing micro/nanospheres using the electrospray technique are shown in Fig. 1-a. Chitosan and alginate core–shell sphere was prepared according to the optimization presented in Table 1, upon different polymer concentrations, flow rates, voltage, and distance. The best parameters for the optimized formulation of the unloaded microsphere were as follows: polymer concentration (2% alginate and 1% chitosan), flow rate (250 µL/h for alginate solution and 150 µL/h for chitosan solution), voltage (25 kV), and distance between the tip of the needle and collector plate (20 cm). Furthermore, different concentrations and pH values of the bovine serum albumin (BSA) as the protein solutions were evaluated to optimize BSA loading into the alginate shell of the microparticle, keeping a round shape (Table supplementary-S1 online). The alginate solution's optimized BSA concentration and pH value were 0.15 mg/mL and 5.5, respectively. Figure 1b shows the schematic reorientation of biological activities of chitosan-alginate core–shell particles.

**Table 1:** Optimization of the chitosan-alginate core–shell particles according to the related parameters.

Run	Chitosan		Alginate		Voltage (kv)	Distance (cm)	Spray condition	Observation
	Concentration (%)	Flow rate	Concentration (%)	Flow rate				
1	1	0.1	2	0.3	20	15	Stable	–
2	1	0.1	2	0.3	20	10	Stable	Large particle in millimeter
3	1	0.1	2	0.3	15	15	Stable	Large particle in millimeter
4	1	0.1	2	0.3	15	10	Stable	Large particle in millimeter
5	1	0.2	2	0.6	20	15	Unstable	Needle clogging
6	1	0.2	2	0.6	20	10	Unstable	Needle clogging
7	1	0.2	2	0.6	15	15	Unstable	Needle clogging
8	1	0.2	2	0.6	15	10	Unstable	Needle clogging
9	1	0.1	3	0.3	20	20	Unstable	Needle clogging

The results of the characterized particles are presented in the following orders:

### Fourier transforms infrared spectroscopy (FTIR) analysis

**Figure 2:**
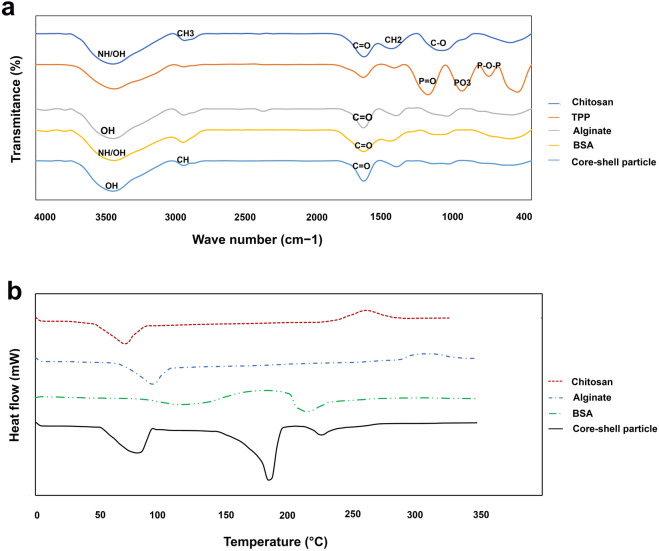
(**a**) FTIR spectra of chitosan, TPP, alginate, BSA, and BSA-loaded chitosan-alginate particle, and (**b**) DSC analysis of chitosan, alginate, BSA and BSA-loaded chitosan-alginate particle.

The FTIR spectra of chitosan, tripolyphosphate (TPP), chitosan particles, alginate, core–shell particles, BSA, and BSA-loaded core–shell particles are illustrated in Fig. 2a. The infrared peaks of chitosan were observed at 3440 cm^−1^, 2920 cm^−1^, 1616 cm^−1^, 1419 cm^−1^, and 1056 cm^−1^, which corresponded to NH and OH stretching, stretching of the methyl group (–CH_3_), C=O stretch of the amide group, aromatic C–H_2_ bending, and C–O stretching, respectively.

For TPP, the major peaks were detected at 1151 cm^−1^, 905 cm^−1^, and 710 cm^−1^, corresponding to P=O stretching, PO_3_ group vibration, and P–O–P bridge asymmetric stretching, respectively.

FTIR spectrum of chitosan particle corresponded to TPP and chitosan spectra, but the absorption bands slightly shifted.

The infrared peaks of alginate were observed at 3440 cm^−1^, 2926 cm^−1^, 1620 cm^−1^, 1383 cm^−1^, and 1013 cm^−1^, which ascribed to the OH stretch of the amide group, –CH_2_ stretching, C=O stretch of the amide group, the asymmetric stretch of C–O–O, and C–O stretching, respectively.

In the core–shell structure, peaks slightly shift from 1616 cm^−1^ and 1419 cm^−1^ to 1631 cm^−1^ and 1381 cm^−1^, respectively. After the complexation of alginate with chitosan, the infrared peak of 2920 cm^−1^ was absent in this compound; the observed changes in the absorption bands of the amino groups, carboxyl groups, and amide bonds can be attributed to an ionic interaction between the carbonyl group of alginate and the amino group of chitosan. The peaks appeared at 3428 cm^−1^, 2920 cm^−1^, and 1631 cm^−1^, attributed to the stretch of OH, CH, and C=O groups in chitosan and alginate components of the core–shell particle.

The major peaks of BSA were observed at 3417 cm^−1^, 2149 cm^−1^, and 1633 cm^−1^, corresponding to the stretch of OH/NH, CO_2_, and C=O groups. The FTIR peaks of BSA-loaded core–shell particles were overlapped with absorption bands of BSA and core–shell particle spectra, in a slightly shifting manner.

### Differential scanning calorimetric (DSC) analysis

Thermograms of DSC results are shown in Fig. 2b. Two endothermic peaks were appeared for BSA at 99 °C and 210 °C. The endothermic and exothermic peaks of 75 °C and 220 °C were appeared for chitosan while the same peaks of 95 °C and 313 °C were observed for alginate thermogram, respectively. Nevertheless, three different endothermic peaks of 74 °C, 183 °C and 227 °C and broader exothermic peak of 340 °C were detected for core–shell microparticles, which can be attributed to the chitosan-alginate complex, calcium chloride, BSA and polymer decomposition, respectively.

### Dynamic light scattering (DLS) and zeta potential analysis

**Figure 3:**
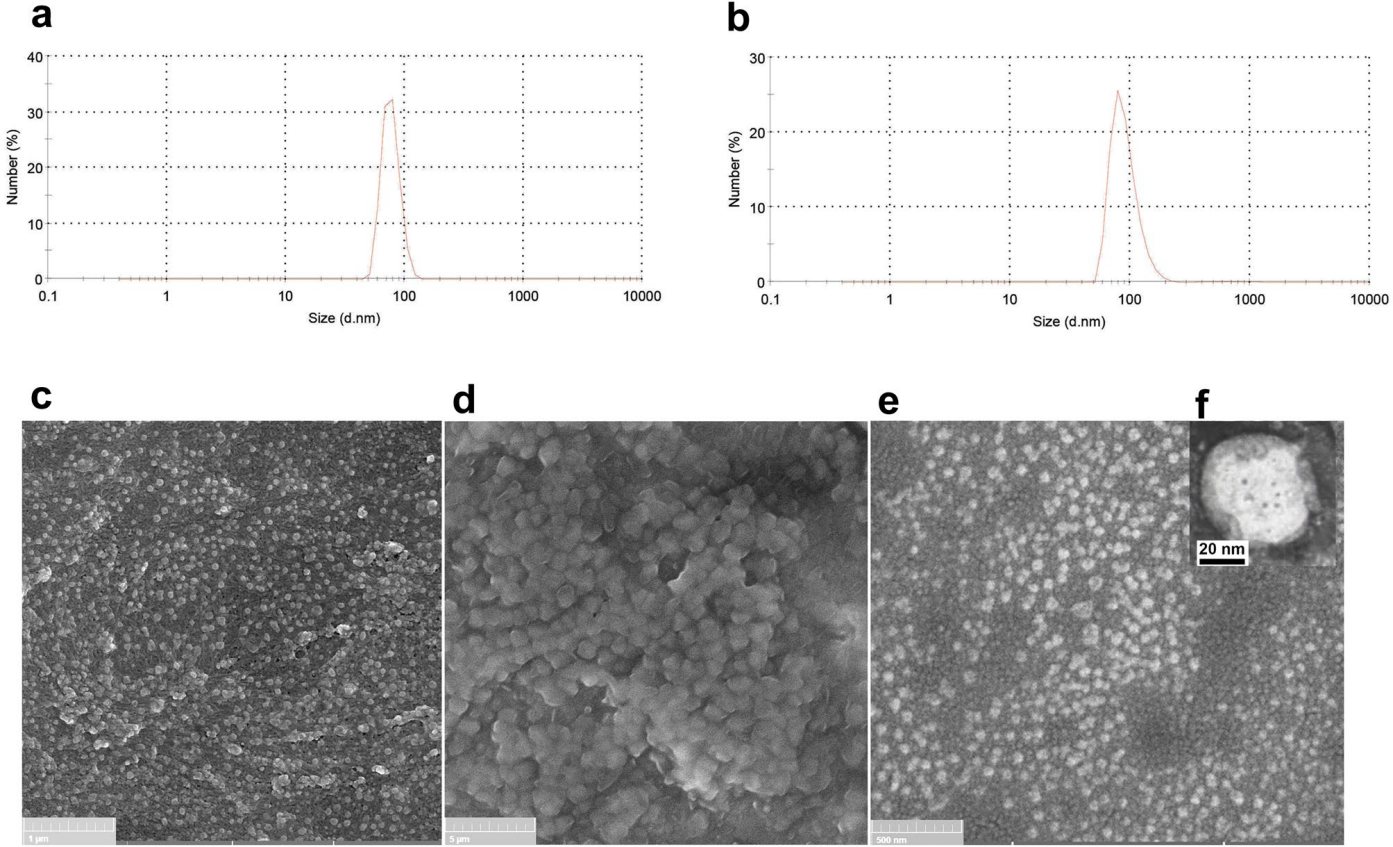
(**a**) DLS graphs of chitosan nanoparticles, (**b**) DLS graphs of chitosan-alginate nanoparticles, (**c**) FE-SEM image of chitosan nanoparticle, (**d**) SEM image of chitosan-alginate microparticles; (**e**) FE-SEM image of chitosan-alginate microparticles; and (**f**) TEM image of the chitosan-alginate nanoparticle.

Figure 3a,b shows the particle size and its distribution results, which were measured using DLS. For the chitosan and chitosan-alginate nanoparticles, the size and PDI of 76.73 nm, and 0.3 and 90.49 nm, and 0.35 were obtained, respectively. In addition, their zeta potential was 43.6 ± 5.08 mV and − 6.35 ± 2.23 mV, respectively.

### Morphology and size measurement using microscopic instruments

Scanning electron microscopy (SEM) images of the prepared particles are shown in Fig. 3c–e; microparticles and nanoparticles exhibited spherical shape and rough appearance. The estimated mean diameter for chitosan nanoparticles, core–shell microparticles and core–shell nanoparticles was estimated to be around 28.78 nm, 1.2 μm, and 50 nm, respectively.

**Figure 4:**
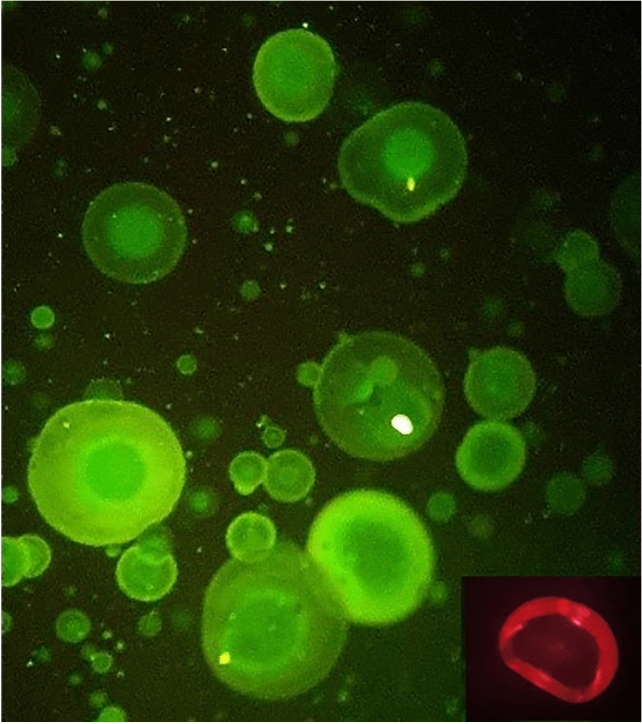
An optical microscope image of fluorescent emission of carboxyfluorecin-stained microparticles. Insert shows the fluorescent emission of Nile-Red-stained microparticles.

Transmission electron microscopy (TEM) image showed more accurate details on the structure of core–shell nanoparticles, with round morphology tending to a quasi-oval shape. The actual size of the core–shell nanoparticles using TEM was estimated to be 31.82 nm, with a shell size of 10.97 nm, confirming the core–shell structure (Fig. 3f). In addition, the core–shell structure was clearly distinguished by the fluorescent microscope in green and red regions of fluorescence emission (Fig. 4).

### Porosity and surface area analysis

**Figure 5:**
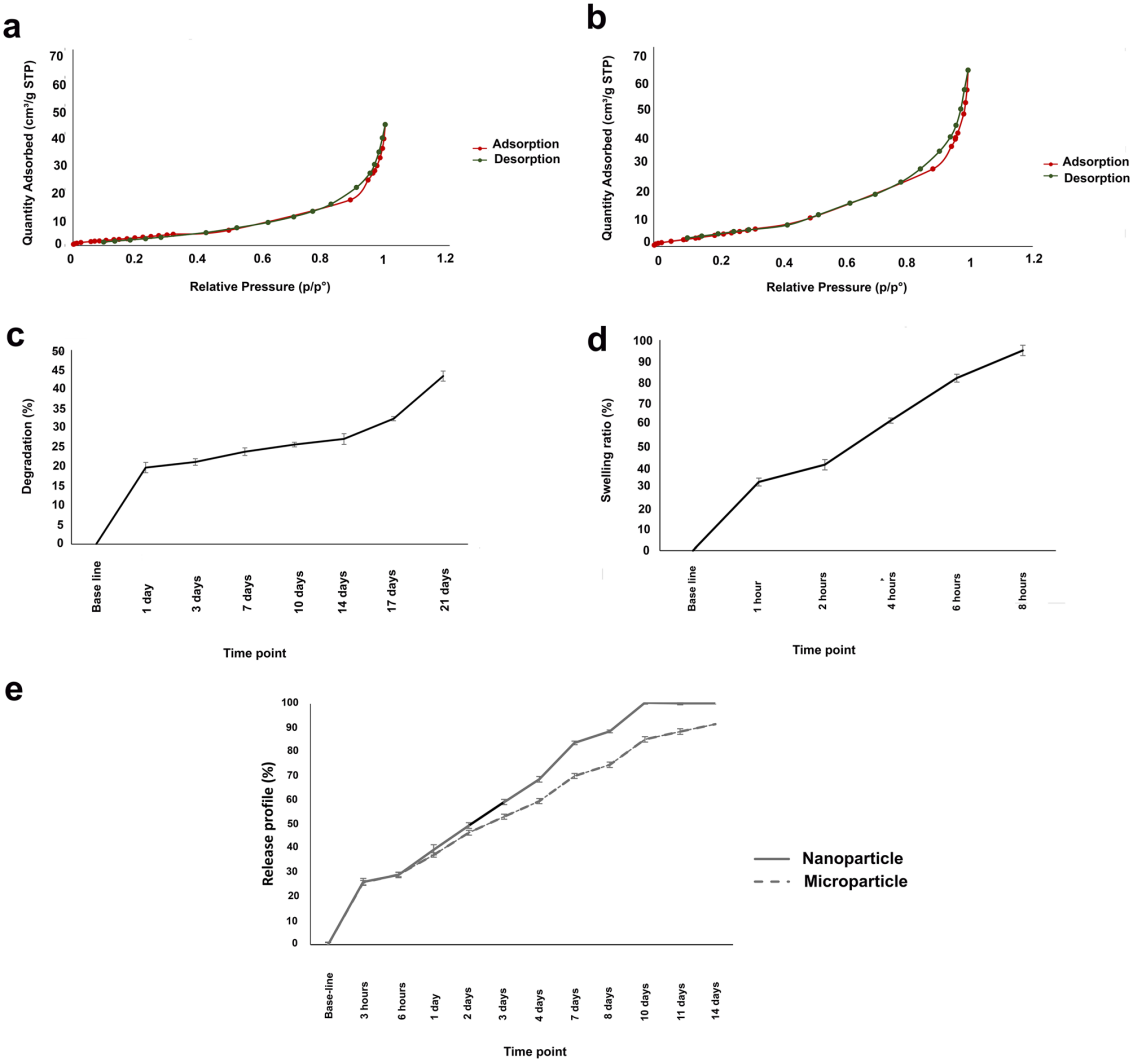
(**a**) N_2_ adsorption–desorption of nanoparticle, (**b**) N_2_ adsorption–desorption isotherms of microparticle, (**c**) Degradation of chitosan-alginate core–shell microparticles at different time points, (**d**) Swelling ratio of chitosan-alginate core–shell microparticles at different time points, and (**e**) Release profile of BSA from BSA-loaded core–shell micro/nanoparticles.

The N_2_ adsorption–desorption isotherm curves for both micro and nanoparticles were are shown at Fig. 5a,b, respectively. The Barrett–Joyner–Halenda (BJH) average pore size, surface area and pore volume for microparticles were about 10.9064 nm, 15.1825 m^2^/g and 0.077 cm^3^/g, while theses values for nanoparticles were about 8.626 nm, 24.915 m^2^/g, and 0.063 cm^3^/g, respectively. The hysteresis loops between adsorption and desorption isotherms were occurred between 0.78 and 0.97 for microparticles and between 0.82 and 0.94 for nanoparticles, respectively.

### Degradation and swelling ratio

The degradation and swelling ratio of the microparticles at different time points are shown in Fig. 5c. 20% of the microparticles were degraded after a day, followed by 45% degradation until 21 days in a slow manner. Moreover, the swelling ratio of the microparticles up to 8 h was investigated (Fig. 5d). As shown, water absorption was increased at different time intervals. In other words, the swelling ratio of about 35% at 1 h was completed after 8 h of water exposure (about 98%).

### Loading estimation and protein release profile

According to bicinchoninic acid (BCA) assay results, the loading content of micro and nanoparticles were 1.21% and 1.65%, while their encapsulation efficiency was calculated to be 74.78% and 97.61%, respectively. In addition, the cumulative release profiles for BSA-loaded core–shell micro/nanoparticles are presented in Fig. 5e.

A burst release of around 14.61% and 16.76% was discovered for microparticles and nanoparticles in the first 30 min, reaching about 55.04% and 49.74% from the loaded amount after 1 day, respectively. The 90% BSA release was observed after 14 days for microparticles and 10 days for nanoparticles. The release profiles showed a sustained delivery manner.

### Hemolysis

**Figure 6:**
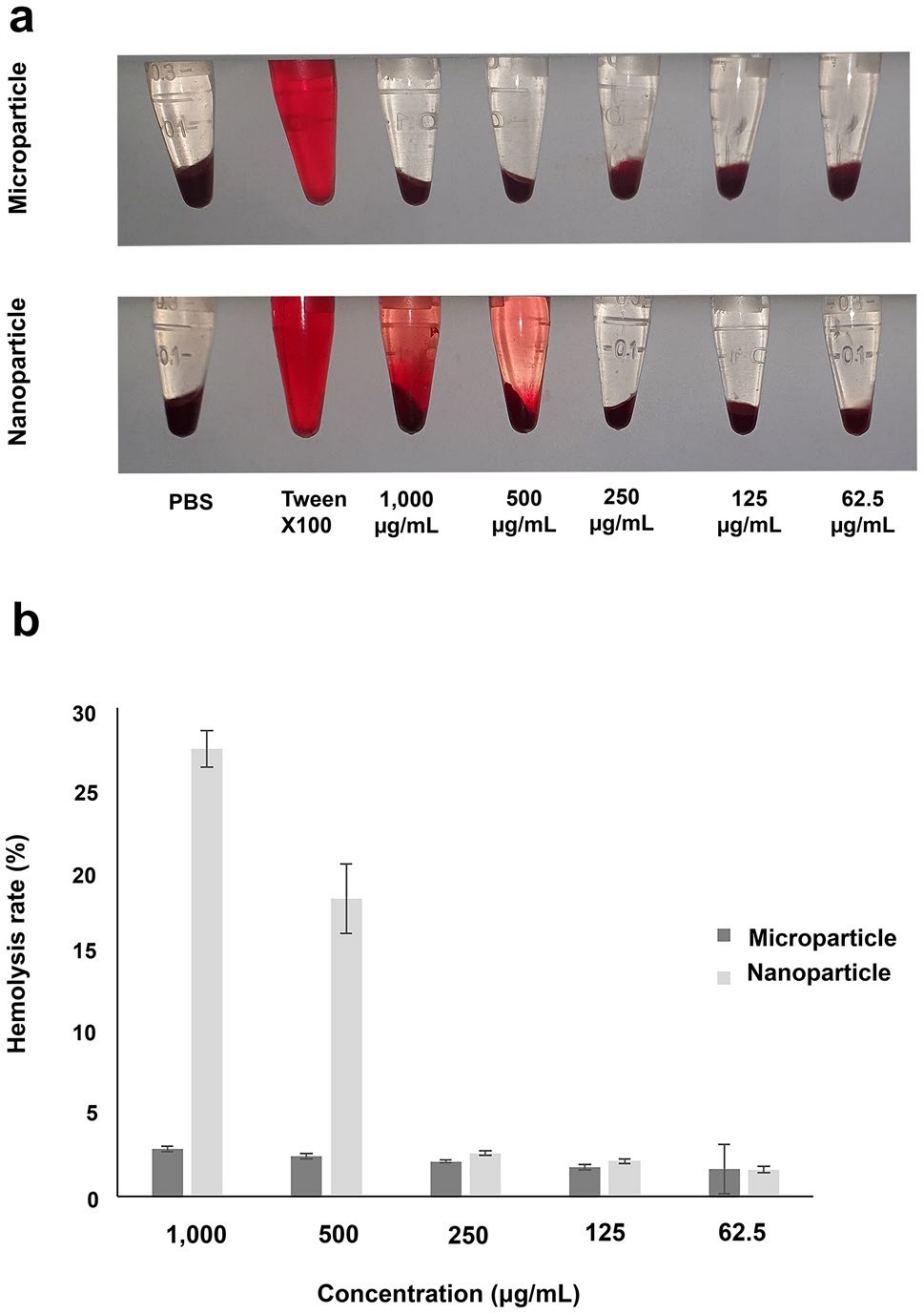
(**a**,**b**) Hemolytic activity of core–shell micro/nanoparticles at different concentrations.

Treatment of diluted erythrocytes with micro/nanoparticles at different concentrations, as well as Triton-X-100 (positive control) and phosphate buffer saline (PBS) (negative control), are shown in Fig. 6a,b. For microparticles, all concentrations showed an excellent hemolytic activity. However, for nanoparticles, acceptable hemocompatibility was observed only for concentrations equal to or less than 250 μg/mL, similar to the results obtained for the biocompatibility assay.

### Protein corona

**Figure 7:**
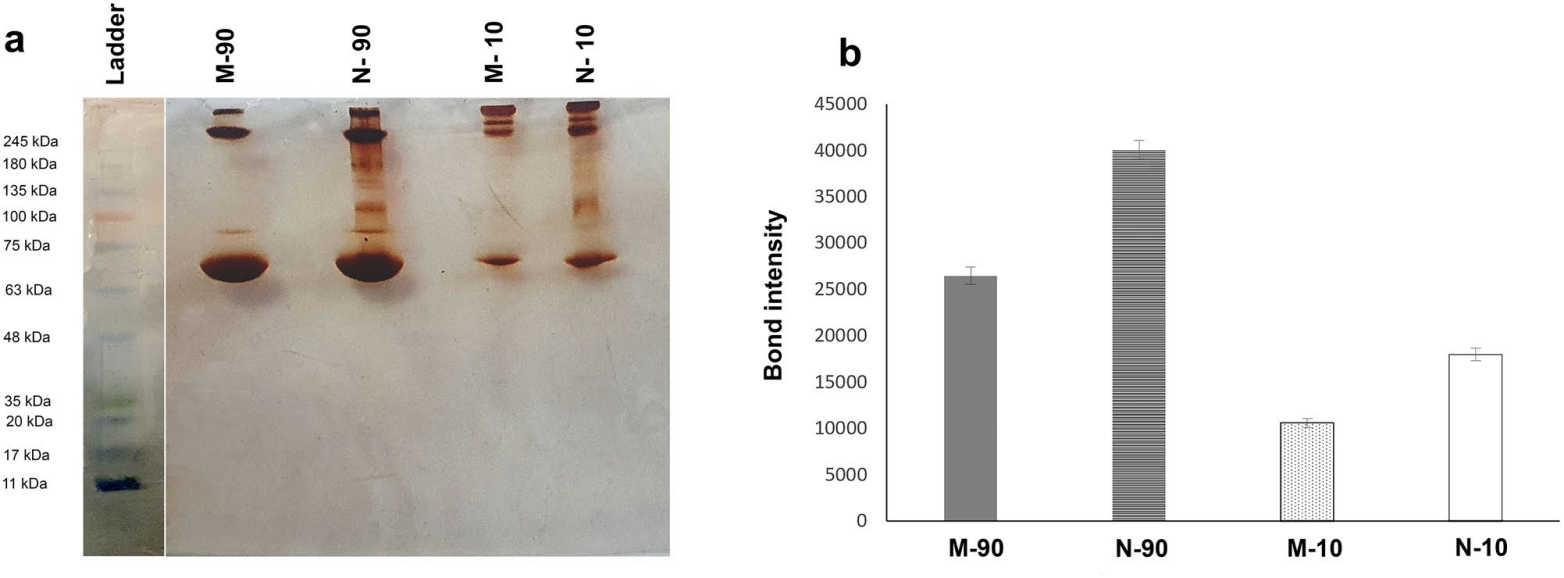
(**a**) SDS-PAGE gel (12%) of the whole (90%) and diluted (10%) human plasma proteins obtained from microparticles and nanoparticles. The molecular weight of the proteins in the standard ladder is reported for reference, (**b**) histograms representing the total band intensity of proteins associated with micro/nanoparticles after exposure with 90- and 10% human plasma (Bond intensity indicates the concentration of proteins that bind to particles).

The intensity of protein bonds absorbed by the micro/nanoparticles is shown in Fig. 7a,b. When micro/nanoparticles were treated with whole plasma, greater protein absorption was observed in nanoparticles than in microparticles (1.51-fold change). In the case of treating micro/nanoparticles with diluted plasma, the same results were obtained (1.69-fold change). However, the extent of the absorbed protein was greater in whole plasma. The observation was related to band intensities higher than 63 kDa, while no protein bands were detected below the molecular weight.

### Cytotoxicity

**Figure 8:**
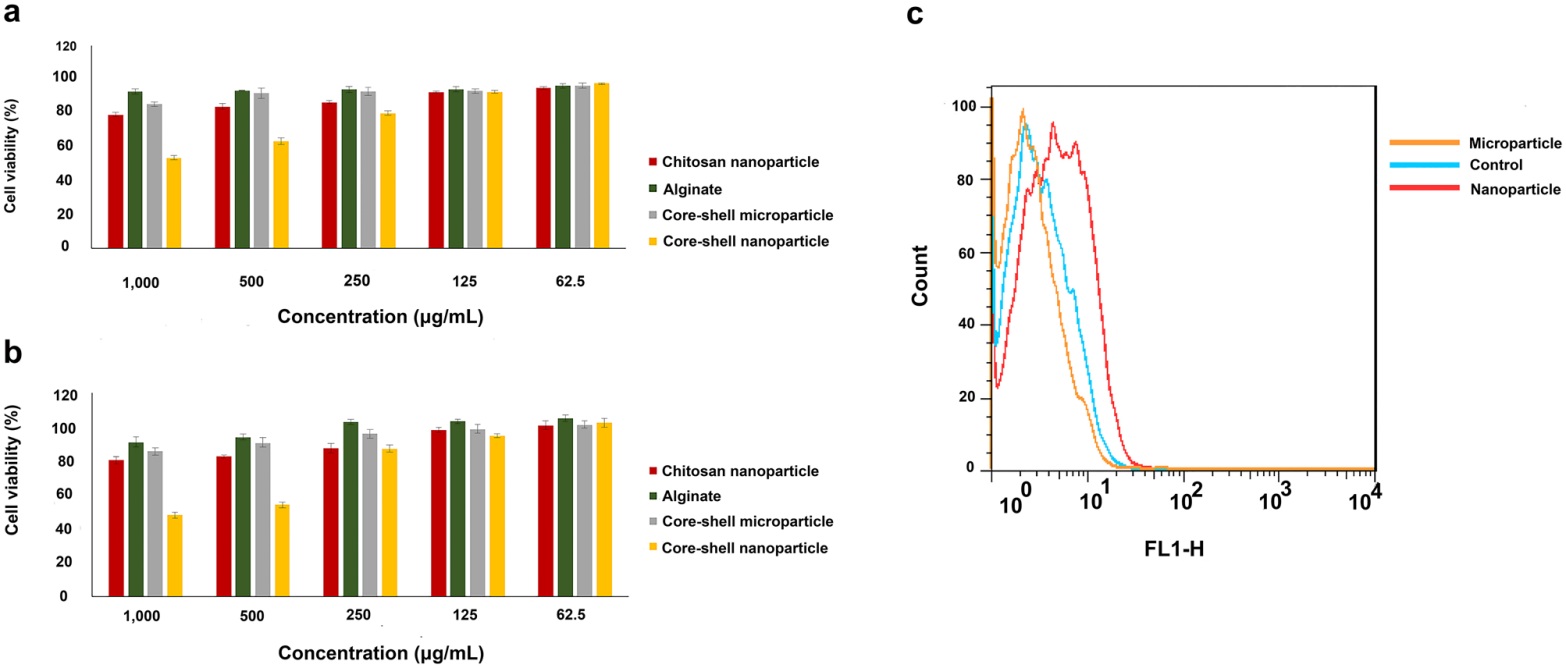
(**a**) Cell viability of micro/nanoparticles at 24 h; (**b**) cell viability of micro/nanoparticles at 72 h and (**c**) cellular uptake of Nile-red from fluorescence-labeled core–shell micro/nanoparticle by flow cytometry.

Cells were treated with different concentrations of chitosan nanoparticles, alginate, and core–shell particles for 24 and 72 h (Fig. 8a,b). For core–shell microparticles, at all concentrations, more than 70% cell viability was observed at all time points. For core–shell nanoparticles, cell viability decreased to 53.75 and 63.69 for 24 h treatment and 48.06 and 54.05 for 72 h incubation in 1000 and 500 μg/mL concentrations, respectively, indicating cell toxicity more than microparticles. However, as shown in Fig. 8b, the desired cell viability was obtained at concentrations equal to or less than 250 μg/mL up to 72 h. No toxicity was observed for chitosan and alginate polymer. The IC50 value was possible to calculate just for 72 h core–shell nanoparticles exposure, which was 332.76 μg/mL.

### Cellular uptake

The internalization of the particles into the adipose stem cells after 6 h was evaluated using flow cytometry. Because the Nile red in free form and loaded particles emit strong red fluorescence, it can be used to quantify cellular uptake. As shown in Fig. 8c, an increase in the cellular uptake efficiency of up to about 4 folds was observed for the nanoparticles rather than the microparticles, indicating improved cell internalization in nanospheres. Cells without Nile red, which showed autofluorescence, were considered as the negative control.

## Discussion

Nowadays, scientists have been interested in designing the core–shell particles to provide a sustained delivery system for biomedical applications^9,20,21^. In this regard, the fabrication of protein-loaded core–shell particles using the electrospraying technique would remove major obstacles related to sequential accessible/available protein considerations for cellular differentiation. The protein-loaded core–shell spheres prepared by the method have many advantages, such as the prolonged lifetime of drug release, suppressed burst release, and the protection of the protein from harsh environments^5^. The present study used the novel protein loading procedure by coaxial electrospraying to fabricate micro/nanoscales of the core–shell particle sizes, separated through a centrifugation procedure.

Logically, the selection of polymers having opposite charges in core and shell compartments facilitates polyelectrolyte complexation through electrostatic interactions between them. Hence, chitosan as a positive charge polymer and alginate as a negative one were chosen for coaxial electrospraying of micro/nanoparticles. Interestingly, considering alginate in the outer layer gave an opportunity to cross-link the polymer using calcium chloride, assuring sustained delivery of the loaded materials. In this regard, BSA was used as a model protein.

Experimentally, different parameters (i.e., polymer and protein concentration, flow rate, voltage, and distance) were optimized to gain micro/nanoparticles with desired size and morphology. Studies have reported that the alginate to chitosan weight ratio may influence the physical properties of core–shell nanoparticles. Manipulation in core–shell polymer ratio is limited upon identity and consideration of material for loading. In our study, the alginate (2%) to chitosan ratio (1%) was optimized to be 2:1 (w/w), and more limitation was faced in manipulating BSA concentration (0.15 mg/mL) for loading in the shell part.

Various alginate to chitosan ratio has been reported in the literature to produce core–shell particles, which can be attributed to distinct preparation techniques, different electrospraying parameters such as needle diameter, and identify of materials considered for loading (i.e., naringenin, insulin, and transforming growth factor-Betha/dexamethasone)^22–25^. However, to allow stable co-flow avoiding needle clogging, we could not further manipulate the viscosity of the alginate and chitosan upon concentration, confirming the study of Williams et al.^26^.

It has been reported that the optimum flow rate of electrospraying particles were 0.5- and 0.6 mL/h for chitosan and alginate solution, respectively^25^. In comparison, we could only use a flow rate of 0.1 mL/h and 0.3 mL/h for chitosan nanoparticles and alginate polymer, respectively, preventing the sphere formation on a millimeter-scale.

In addition, we found that the concentrations of BSA in alginate solution have a direct impact on particle shape. In other words, increasing the concentration of BSA in alginate led to the formation of tailed particles.

In FTIR spectra of core–shell nanoparticles, not only no newly identified peaks were identified, but also all absorption bands were corresponded to the ingredients, indicating no covalent interference of BSA with polymers and materials. However, appearance a broader endothermic peak (∼ 74 °C) in DSC thermogram of core–shell microparticles between the peaks of pure chitosan and alginate showed physical interaction of polymers. Furthermore, a little shift in second peak of BSA thermogram in DSC analysis of the particle indicates electrostatic interaction among protein and two polymers. However, BSA has conserved its crystalline state in the core–shell particles.

The size of nanoparticles measured by the DLS technique (∼ 90 nm) was larger than the size determined by TEM (50 nm), attributed to the hydrodynamic radius measured by the former rather than the actual size utilized by the latter technique. In the literature, in line with our study, the slight difference in measured sizes between DLS and TEM/SEM has been reported with other nanomaterials^27,28^.

Particle size is an important physical property that directly affects the release and biological fate of the encapsulated bioactive compound^17,29^. Using a centrifugation procedure, two different particle sizes in the nano- and submicron/microscale were able to be produced.

According to the dissolution profile of BSA release from the core–shell micro/nanoparticles, the burst release based during the initial 3 h was continued by sustained release trend until 14 days. However, the release rate from the core–shell nanoparticles was faster than that from the microparticles, as the complete release occurred on the 10th day compared to that from the microparticles (14th day). Logically, the slightly faster protein release from nanoparticles can be attributed to the higher surface area in the nanoscale, leading to increased interaction with dissolution media and enhancing water permeation. Some studies have reported drug release (i.e., insulin and naringenin) from core–shell chitosan-alginate particles, indicating the sensitivity of the core–shell to the pH of media for oral delivery application^22,24^. In contrast, the sequential sustained drug release from core–shell particles was just investigated in physiological pH for tissue engineering purposes. For example, transforming growth factor-βetha 1 and dexamethasone were released sequentially for 2 weeks, ensuring the required concentrations for cell differentiation^23^. It is worth noting that our study approach is close to the second one. Interestingly, a direct rational relationship was observed among swelling ratio, degradation percentage, and drug release profile. We believe that drug release kinetics can be tuned depending on the needs by modifying the particle size by having the same polymer.

The acceptable hemolytic activity of nanomaterials has been considered critical before their clinical application in tissues in contact with blood. According to the hemolysis assay, excellent hemocompatibility was observed for core–shell microparticles and in specific concentrations of nanoparticles (below 500 μg/mL), covering the concentration range used in biological tests.

Furthermore, the biocompatibility of nanomaterials is also important. Therefore, the cytotoxicity investigation for stem cells exposed to different concentrations of core–shell particles was performed in the second step. The cytotoxicity results were in good conformity with the hemolytic results for microparticles and nanoparticles. However, more biocompatibility resulted from cell viability evaluation for microparticles than nanoparticles. Apparently, significant toxicity has been observed after 72 h for core–shell nanoparticle treatments of adipose stem cells at concentrations outside the studied range (more than 500 μg/mL), as reached to IC50 value (332.76 μg/mL). Our findings confirmed the results reported by Nguyen K.T et al., in which more toxicity was observed for the nanoparticle platform of poly-nisopropylacrylamide spheres than for microparticles^17^.

Some studies have shown that the size of the particles determines their biological fate. Indeed, besides the charge, surface chemistry, and shape, particle size is one of the critical parameters affecting biological activities such as biocompatibility^30^, cellular uptake^31^, and protein absorption^32^. In this regard, our microparticles showed more desired hemo/biocompatibility than nanoparticles, confirming the size-dependent biological fate. Furthermore, in our study, we observed that core–shell nanoparticles were internalized more efficiently by adipose stem cells than microparticles, which further supports our conclusion. Logically, more interaction in the bio-interface for nanoparticles due to increased surface area has been resulted in more cellular uptake and subsequently, different hemolytic activity as well as cell viability. However, the cellular uptake and toxicity are reported to be dependent on the cell type, exposure time, and particle concentration. For example, it has been reported that cellular uptake of nanospheres is dose and time-dependent in both endothelial cells and smooth muscle cells. Interestingly, it has been reported immune cells have a higher affinity to microspheres than nanospheres in contrast to vascular cells. The biological findings have a straight correlation with release patterns in each scale of micro/nanoparticles as more release was observed for the nanoscale platform, confirming the results of the reported study^33^.

Due to the same reason, more surface area in nanoscale form and more protein absorption was observed for nanoparticles than microparticles after plasma treatment, as identified by SDS-PAGE. The results are in accordance with some previous reports^32,34^. The study of protein adsorption pattern is also important, because it determines the cell adhesion, immune response, tissue healing and remodeling^35,36^. Furthermore, faster protein release from nanoparticles can be attributed to their higher surface area to volume ratio. The higher surface area for nanoparticles than microparticles in a core–shell platform was confirmed by the results of Brunauer–Emmett–Teller (BET) analysis. However, the existence of a hysteresis loop between adsorption and desorption isotherms reflected the pores with different sizes/structures. At the region of relative pressure (P/P0) of more than about 0.8, the isotherms were increased rapidly, indicating agglomeration phenomenon or developing larger particles. Generally, both isotherm curves are similar to type IV adsorption, according to IUPAC classification.

Each nanoparticle or microparticle has its own advantages and disadvantages, and its choice is dependent on its application. The microspheres can encapsulate larger amounts of drugs. Although they cannot be easily used for intravenous or systemic delivery, as they may agglomerate and cause clotting, they are effective for local delivery, such as subcutaneous injection, and can be used in sustained-release systems. In comparison, inflammation response and foreign body reaction were considered challenges in developing a high diameter microsphere. As we mentioned in our study, a microsphere’s greater hemocompatibility suggests it could be applied in tissues in contact with blood. Furthermore, according to our results, the microsphere can be considered a suitable drug delivery system when more sustained delivery is needed. However, the major drawback of microsphere utilization is lower cellular uptake than nanoparticles. We suggest the protein-loaded core–shell microsphere as the powerful carrier for local treatment in regenerative medicine.

As the particle size becomes smaller, the surface area to volume ratio is increased, implying more interaction in the bio-interface and subsequent cellular uptake. This feature of the nanosphere introduces it as a favored drug delivery system when more efficacy and pharmacodynamic improvement are needed. However, as a major concern, suitable concentrations must be selected to avoid cellular toxicity as well as hemolytic activity. The core–shell nanoparticle facilitates not only intravenous administration but also local delivery.

However, as the protein release from the nanoparticles is faster than that from the microparticles, the correlation between the critical concentration of release protein and cell differentiation time should be considered. It is noteworthy that increased protein corona profiles on the nanoparticle surface may have controversial effects, as the corona composition can navigate particles to the target site. Therefore, further analysis by mass spectrometry should be performed to identify protein composition.

Taken together, two developed core–shell nanoparticles and microparticles with individual advantages upon size can be used for biomedical application and regenerative medicine, considering their effects on bio/hemocompatibility, cellular uptake, and extent of protein absorption.

## Conclusion

Fabricating core–shell particles in different-sizes can provide sustained release protein-based delivery systems for biomedical applications. Here, we have shown that coaxial electrospraying is a favorite technique for developing desired delivery systems for protein release. The well characterized vehicles in nano/micro scales have different drug release profiles, hemo/biocompatibility, cellular uptake, and protein corona profile. The development of such biodegradable micro and nanoparticles gives an opportunity to select a special size with individual biological activities for local and systemic protein delivery and cell differentiation in regenerative medicine.

## Methods

### Materials

Medium viscosity sodium alginate (80,000–120,000 Da), low molecular weight chitosan (50,000–190,000 Da), TPP, calcium chloride, acetic acid, Nile-red, carboxyfluorescein, silver nitrate, and PBS were purchased from Sigma-Aldrich (St Louis, MO, USA). BSA and BCA protein assay kit was obtained from Thermo Fisher Scientific Inc. (Waltham, MA, USA).

Human adipose stem cells were obtained from the Research Institute of Dental Sciences, Shahid Beheshti University of Medical Sciences, Iran, Tehran. 3-(4,5-Dimethylthiazol-2-yl)-2, 5-diphenyltetrazolium bromide (MTT), and Dimethyl sulfoxide (DMSO) were purchased from Sigma-Aldrich Chemicals (Germany). Dulbecco’s Modified Eagle’s Medium (DMEM), Fetal Bovine Serum (FBS), and Penicillin–Streptomycin 1% were obtained from Gibco (Grand Island, NY, USA). All aqueous solutions were prepared using double distilled water. All materials for Sodium Dodecyl Sulfate Polyacrylamide Gel Electrophoresis (SDS-PAGE) were prepared as previously reported. All reagents used were of analytical grade.

### Synthesis of nano- and microparticles

Chitosan-alginate core–shell nano- and microparticles were prepared using the coaxial electrospray method.

### Preparation of electrospray solution

The core solution containing chitosan was prepared using the ionic gelation method. Briefly, chitosan solution (3.75 mg/mL) was prepared by dissolving chitosan powder in acetic acid solution 2% (v/v) and stirred overnight. The solution was then passed through a 0.20 μm filter membrane to remove any non-dissolved particles. Next, TPP solution (2.5 mg/mL) was further added dropwise to the emulsion and stirred for another 2 h. For the shell solution, sodium alginate solution (10 mg/mL) was prepared by dissolving alginate powder in double deionized water and stirred overnight.

For the core part, BSA solution (1 mg/mL) was added to chitosan before the incorporation of TPP, while for the shell part, BSA solution (10% w/w alginate powder) was prepared, and the pH was adjusted to 5.5 with 0.01 M NaOH and added to alginate solution.

### Electrospray procedure

The prepared alginate and chitosan solution was transferred into the syringes connected to a coaxial needle and kept perpendicular to the collector plate. Two syringe pumps are independently connected to the coaxial needle, which consists of two concentric stainless-steel needles, in which the diameter for the outer and inner needles were 19 and 26 gage, respectively. Alginate was injected into the outer syringe, while chitosan was injected into the inner syringe. To obtain a stable Taylor cone jet, the parameters were adjusted according to our preliminary studies (Table 1). The electrospraying was performed toward a cross-linking solution (calcium chloride 3%), with constant stirring at 200 rpm during the procedure.

To separate nanoparticles from the microparticles, electrosprayed particles were centrifuged (2500 rpm/10 min), followed by dialysis of the infiltrated solution against PBS (pH 7.4). Finally, it is transferred to amber glass tubes and stored in the refrigerator until further analysis.

### Preparation of the fluorescence-labeled particles

To examine the core–shell structure of the particles using the fluorescent microscope, the core and shell layers were labeled with carboxyfluorescein and Nile red, respectively. Carboxyfluorescein and Nile-red were prepared at a concentration of 1 mg/mL. A given amount of carboxyfluorescein and Nile-red solution was added to the chitosan and alginate solution, respectively. The solutions were stirred for 3 h in dark conditions. The chitosan solution was cross-linked with TPP, and the labeled micro/nanoparticles were obtained via the electrospraying method as mentioned above.

### Characterization

FTIR analysis was used to examine the chemical structure of the chitosan-alginate particles using the spectrometer (Spectrum Two™ IR; PerkinElmer, Inc., Massachusetts, USA). All spectra were recorded in the range of 400–4000 cm^−1^. A DSC measurement was used to investigate the thermal behavior and molecular status of the particles using universal V4.5A instrument. Briefly, 5 mg of lyophilized powders were transferred into an aluminum pan, at a heating ramp of 10 C/min from 0 to 350 °C under nitrogen purging of 20 mL/min. The empty aluminum pans were considered as the reference group. A DLS spectrometer (Malvern Paralytical Co., Ltd, Malvern, United Kingdom) was used to determine the size and zeta potential of the chitosan and chitosan-alginate nanoparticle. The surface morphology and size of the micro/nanoparticles were observed using SEM (Philips XL30 TMP; F.E.I. Com, Eindhoven, Netherlands) and field emission SEM (MAIA3 TESCAN; Absotec Co., Ltd, Bangkok, Thailand), respectively. The synthesized nanoparticles' morphology and core–shell structure was investigated using TEM (EM10C-100 kV; Carl Zeiss Meditec AG, Jena, Germany). The core–shell structure of the bilayer carriers was also confirmed using fluorescent microscopy (TCM 400, Labo America Inc, Fremont, CA, USA). For porosity and surface area analysis, the specific surface area of the sample was assessed by BET method and the pore size and pore volume distribution was determined by BJH method using the ASAP 2010 static volumetric absorption analyzer (Micromerities Corp., NY, USA). To remove residual moisture on the particles, samples were degassed by heating at 110 °C for 2 h before nitrogen treatment as the absorbent^37^.

### Swelling index

The swelling index of microparticles was measured by immersing dry microspheres (5 mg) in PBS at 37 °C. At predetermined time intervals (1, 2, 4, 6, and 24 h), the microspheres' excess water was removed using the filter paper. The SI of the microspheres was calculated using the following equation:

$$\mathrm{Swelling\, index}= \frac{\mathrm{Weight\,  of\,  swollen\,  microsphere }-\mathrm{ weight \, of\,  dry\,  microspheres}}{\mathrm{weight\,  of\,  dry \, microspheres}}\hspace{0.17em}\times \hspace{0.17em}100$$

### Degradation rate

An accurately weighed amount of 5 mg microparticles (Wa) was immersed in PBS. Samples were incubated at 37 °C while gently shaking. At different time points (1, 3, 7, 10, 14, and 21 days), samples were centrifuged at 10,000 rpm for 20 min. Next, the microparticles were washed twice with distilled water and subsequently lyophilized and weighed (Wb). The degradation rate was calculated according to the following equation:

$$\mathrm{Degradation \, rate}=\frac{\mathrm{Wb }-\mathrm{ Wa}}{\mathrm{Wa}}\times 100$$

### Protein loading estimation

Briefly, BSA-loaded micro/nanoparticles (50 mg) were dissolved in 2 mL PBS and stirred at 500 rpm for 4 h. It was then centrifuged (15,000 rpm/20 min) to precipitate particles. Protein concentration was measured using a BCA protein assay, reading the absorbances by UV/VIS-spectrophotometer (Jasco B/530) at 562 nm (n = 3). The loading capacity of particles was determined as follows:

$$\mathrm{Loading \, capacity }(\mathrm{\%})=\frac{\mathrm{Weight \, of \, measured \, BSA \, in \, loaded \, particle}}{\mathrm{Particle\,  weight}+\mathrm{BSA \, weight}}\times 100$$

$$\mathrm{Encapsulation\,  efficacy }\left(\mathrm{\%}\right)=\frac{\mathrm{Mass \, of \, BSA \, in \, micro}/\mathrm{nanoparticles}}{\mathrm{Mass \, of \, BSA \, in \, the\,  formulation}}\times 100$$

### Protein release from core–shell micro/nanoparticles

In brief, 5 mg particles were dispersed in 1 mL PBS (pH 7.4) and incubated in a shaker incubator (100 rpm/37 °C). The supernatant was collected by centrifugation at various time points (15 min, 30 min, 1 h, 2 h, 3 h, 6 h, 1 day, 2 days, 3  days, 4  days, 7  days, 8  days, 10  days, 14  days, 17  days, and 21  days). The fresh PBS was added after each collection. The BSA concentrations in the supernatant were measured by the BCA Protein Assay versus the obtained absorbance by UV/VIS-spectrophotometer (Jasco B/530) at 562 nm. The cumulative BSA release percentages at each point were calculated according to the standard calibration curve (n = 3).

### Hemolysis

Hemolysis assay was performed against different concentrations of microparticles and nanoparticles (1000, 500, 250, 125, and 62.5 μg) according to previously published procedure (n = 3)^38^. In addition, PBS and Triton X-100 were used as negative and positive controls, respectively. The hemolysis rate was calculated according to the following formula:

$$\mathrm{Hemolysis \, rate}: \frac{absorbance\,  of\,  sample-absorbance\,  of\,  negative\,  control}{absorbance\,  of\,  positive\,  control-absorbance\,  of \, negative \, contol}\times 100$$

According to the criterion reported in the ASTM E2524-08 standard, percent hemolysis < 5% indicates that the test material causes no damage to red blood cells^39,40^.

### Protein corona study

Firstly, fresh human plasma (∼ 100 mL each) was provided from the Iran blood Transfusion Institute, Tehran, Iran. Briefly, plasma was centrifuged (15,000 rpm/4 min) to remove any possible protein aggregate complex as a pellet. The accurate volume of 0.1 mL of each micro/nanoparticles (1 mg/mL) was added to 0.9 mL of human blood plasma and incubated for 1 h at 37 °C. The procedure was repeated for diluted plasma (10%) (n = 3). To investigate the hard corona profile, the particles were centrifuged for 10 min at 10,000 rpm and 15,000 rpm for microparticles and nanoparticles, respectively. The procedure was followed by resuspending the pellet in PBS (0.5 mL) and washing it in the same buffer 3 times. The protein corona on the surface of the particles was identified using the standard SDS-PAGE and silver nitrate staining, according to the reported method^27^. The protein bands’ intensity was analyzed by Image J software (1.410 version).

### Cell viability

The cell viability of the chitosan nanoparticles, alginate, and core–shell micro/nanoparticles was assessed by MTT assay (Sigma-Aldrich Chemicals, Germany). Briefly, adipose stem cells were seeded into a 96-well plate (1 × 10^4^/well) in DMEM supplemented with 10% FBS (Gibco, Australia), and Penicillin–Streptomycin 1% (Gibco, Australia) and incubated at 37 °C. After 24 h, the medium was replaced with 100 µL refreshed medium containing different concentrations of prepared chitosan nanoparticle, alginate, and core–shell micro/nanoparticles (1000, 500, 250, 125, and 62.5 μg/mL). The cells cultured in a growth medium were served as control. Three replicates were performed for each group. After 24- and 72 h, the cells were treated with MTT reagent for 4 h at 37 °C. Next, the MTT solution was replaced with 100 µL of DMSO solvent (Sigma-Aldrich Chemicals, Germany) to dissolve the formed formazan. The absorbance of each well was measured at 570 nm using an ELISA Reader (Anthos 2020, Austria). The cells viability was calculated using the following equation:

$$\mathrm{Cell\,  viability }(\mathrm{\%}) =\frac{absorbance\,  of\,  test}{absorbance \, of\,  control}\times 100$$

### Cellular uptake

Cellular uptake of micro/nanoparticles was investigated by using fluorescent Nile red embedded in the particles. All the procedures were performed in a dark setting. Human adipose stem cells were seeded in a 6-well plate (1 × 10^5^ cells/well) in a culture medium. After 24 h, the media was replaced with the culture medium containing 125 µg/mL micro/nanoparticles and incubated at 37 °C and 5% CO_2_. After 6 h, the cells were washed three times with PBS buffer, and the red-emitting light (excitation wavelength, 559 nm; emission wavelength, 635 nm) of Nile-red was measured by flow cytometer (FACScan, LYSIS II, Becton Dickinson).

## Supplementary Information

Supplementary Information.

## Data Availability

The datasets generated during and/or analyzed during the current study are available from the corresponding author on reasonable request.
