# Supplementary material for: Size-dependent bioactivity of electrosprayed core–shell chitosan-alginate particles for protein delivery
Source: Sci Rep. 2022 Nov 22;12:20097. doi: 10.1038/s41598-022-24389-x (PMC9684514; doi:10.1038/s41598-022-24389-x)
Supplement: Supplementary file 1 — Supplementary Information. [file 41598_2022_24389_MOESM1_ESM.pdf]

Table supplementary 1: Optimization of Bovine Serum Albumin loading into alginate shell of the particle.

Run	BSA concentration (mg/ml)	pH	Particle shape after spraying in calcium chloride
1	1	3	Tailed shaped
2	1	5.5	Tailed shaped
3	0.5	3	Tailed shaped
4	0.5	5.5	Tailed shaped
5	0.25	3	Tailed shaped
6	0.25	5.5	Tailed shaped
7	0.15	3	Tailed shaped
8	0.15	5.5	Round shaped

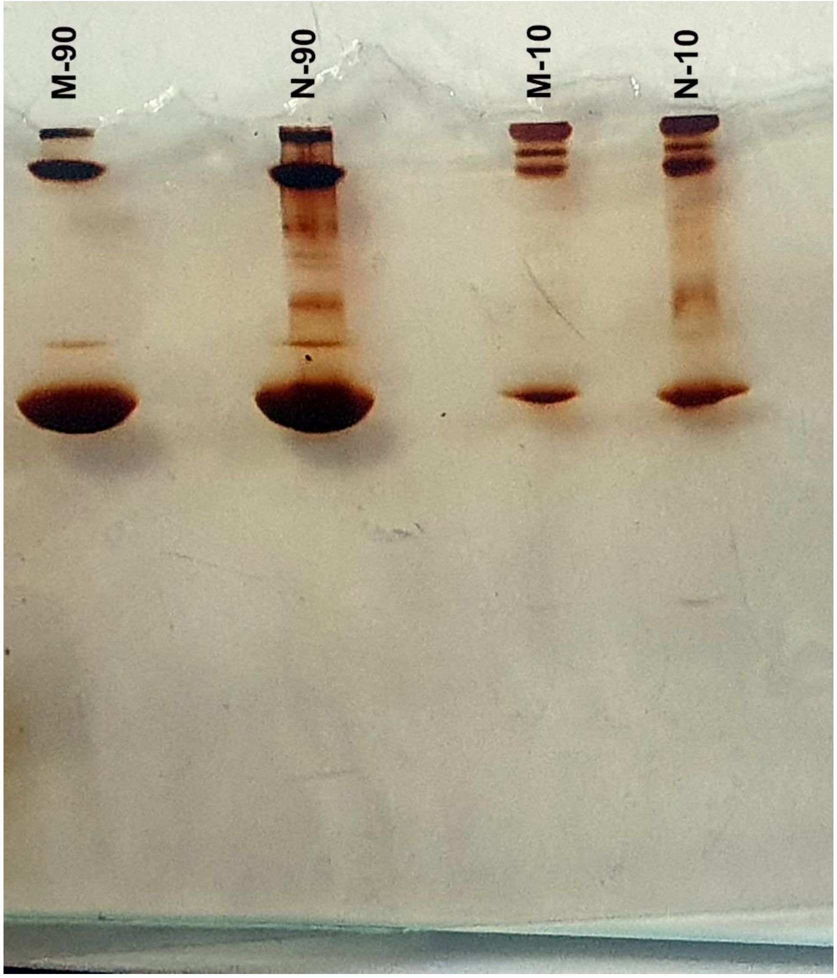

Figure supplementary 1: Non-cropped SDS-PAGE gel of the whole (90%) and diluted (10%) human plasma proteins obtained from microparticles (M-90 and M-10) and nanoparticles (N-90 and N-10).
